# Dual-Energy Computed Tomography in Urological Diseases: A Narrative Review

**DOI:** 10.3390/jcm13144069

**Published:** 2024-07-11

**Authors:** Andrea Coppola, Luigi Tessitore, Federico Fontana, Filippo Piacentino, Chiara Recaldini, Manuela Minenna, Paolo Capogrosso, Roberto Minici, Domenico Laganà, Anna Maria Ierardi, Gianpaolo Carrafiello, Fabio D’Angelo, Giulio Carcano, Laura Maria Cacioppa, Federico Dehò, Massimo Venturini

**Affiliations:** 1 Diagnostic and Interventional Radiology Unit, Circolo Hospital, ASST Sette Laghi, 21100 Varese, Italy; 2 Department of Medicine and Technological Innovation, Insubria University, 21100 Varese, Italy; 3 Urology Unit, Circolo Hospital, ASST Sette Laghi, 21100 Varese, Italy; 4 Radiology Unit, Dulbecco University Hospital, 88100 Catanzaro, Italy; 5 Department of Experimental and Clinical Medicine, Magna Graecia University of Catanzaro, 88100 Catanzaro, Italy; 6 Radiology Unit, IRCCS Ca Granda Ospedale Maggiore Policlinico, Via Sforza 35, 20122 Milan, Italy; 7 Department of Medicine and Surgery, Insubria University, 21100 Varese, Italy; 8 Orthopedic Surgery Unit, ASST Sette Laghi, 21100 Varese, Italy; 9 Emergency and Transplant Surgery Department, ASST Sette Laghi, 21100 Varese, Italy; 10 Department of Clinical, Special and Dental Sciences, University Politecnica delle Marche, 60126 Ancona, Italy; 11 Division of Interventional Radiology, Department of Radiological Sciences, University Hospital “Azienda Ospedaliera Universitaria delle Marche”, 60126 Ancona, Italy

**Keywords:** kidney neoplasms, urinary bladder neoplasms, ureteral neoplasms, polycystic kidney diseases, urinary tract stones, lymph node metastasis, dual-energy computed tomography, iodine density, effective atomic number, virtual monoenergetic imaging

## Abstract

Dual-Energy computed tomography (DECT) with its various advanced techniques, including Virtual Non-Contrast (VNC), effective atomic number (Z-eff) calculation, Z-maps, Iodine Density Index (IDI), and so on, holds great promise in the diagnosis and management of urogenital tumours. In this narrative review, we analyze the current status of knowledge of this technology to provide better lesion characterization, improve the staging accuracy, and give more precise treatment response assessments in relation to urological tumours.

## 1. Introduction

Urological cancers include a group of tumours that impact the organs within the urinary system and the male reproductive system, such as the prostate, bladder, kidneys, testicles, and urethra. Managing these conditions requires thorough diagnostic assessments and the suitable treatment strategies due to their considerable effects on patient well-being and the quality of life [1,2].

Imaging represents a cornerstone in both the study and treatment of urological cancers. Among the forefront diagnostic instruments employed is Dual-Energy Computed Tomography (DECT). This sophisticated imaging method harnesses two distinct X-ray energies to capture the highly detailed and precise images of the tissues and lesions within the urological organs, enhancing diagnostic accuracy [3].

This pioneering approach empowers healthcare providers to identify lesions at an earlier stage, conduct more comprehensive assessments of their characteristics, define better diagnostic and therapeutic strategies, and target the therapeutic interventions. DECT stands as a remarkable stride in the realm of diagnosing and monitoring urological cancers, significantly enhancing the treatment outlooks and elevating the quality of life for the individuals grappling with these intricate diseases [4].

DECT operates on the principle of how X-rays interact diversely with tissues at varying energy levels. This disparity yields supplementary insights into the chemical and physical makeup of tissues, enhancing the distinction among diverse anatomical structures. In DECT, images are captured using two different X-ray energies either in rapid succession or simultaneously, yielding dual datasets that enable in vivo X-ray spectroscopy. Consequently, this method allows the generation of composite images or tailored analyses. For instance, the effective atomic number (Z-eff) technique scrutinizes the predominant atomic number within a voxel, providing crucial data about tissue’s chemical composition. It aids in identifying calcifications, iodine, or other substances within tissues. From Z-eff, maps such as Prevalent Atomic Number (Z-Maps) and Iodine Density Index (IDI) can be derived, offering insights into the distribution of the predominant atomic number and iodine in the body, particularly valuable in assessing vascular lesions and characterizing tumours. Virtual Non-Contrast (VNC) imaging, a DECT method, generates CT images resembling non-contrast scans from contrast-enhanced CT scans. This is beneficial for patients unsuitable for radiation exposure (such as young or pregnant individuals) or when a non-contrast assessment is necessary post-acquisition. With virtual monoenergetic imaging (VMI), the polyenergetic X-ray spectrum (comprising high and low kVp data) is processed to reconstruct images at a selected hypothetical energy level within a range of 40–140 keV. Lower keV emphasizes the enhancement (beneficial for oncological and vascular applications), while higher keV reduces the noise, including mitigating beam-hardening artefacts [5].

The advancements in DECT and dual-layer spectral CT eliminate the need to predefine spectral reconstructions before the acquisition process, offering added convenience and flexibility [6,7].

In essence, Dual-Energy Computed Tomography (DECT) offers numerous advantages, encompassing enhanced diagnostic precision, the improved distinction between normal and pathological tissues, the capability to acquire contrast-free images, and the potential to analyze tissue’s chemical composition. Nevertheless, it is crucial to acknowledge that utilizing DECT necessitates specialized equipment and proper training for healthcare professionals. Furthermore, specific applications may vary based on the clinical requirements. This review aims to delve into how spectral CT functions as an inventive asset in combating urological cancers. It provides an outline of its key applications and its influence on diagnosing and treating these conditions.

## 2. Materials and Methods

**Figure 1:**
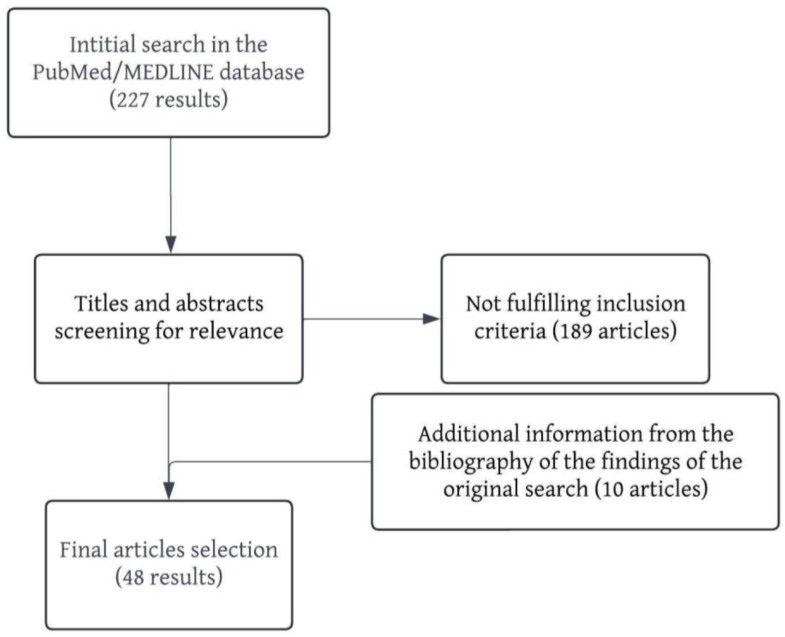
Flowchart of the article selection process.

For the literature search in the PubMed/MEDLINE database, the search term was: (“DECT” AND “urology”) OR (“DECT” AND “kidney”) OR (“DECT” AND “ureter”) OR (“DECT” AND “bladder”) OR (“DECT” AND “prostate”) OR (“DECT” AND “pelvic nodes”) OR (“SPECTRAL CT” AND “urology”) OR (“SPECTRAL CT” AND “kidney”) OR (“SPECTRAL CT” AND “ureter”) OR (“SPECTRAL CT” AND “bladder”) OR (“SPECTRAL CT” AND “prostate”) OR (“SPECTRAL CT” AND “pelvic nodes”). This search was supplemented with additional information from the bibliography of the findings of the original search. Only articles that were published in English were selected. Two authors independently screened the titles and abstracts for relevance. In the case of discrepancy, the judgement of a third author was requested. After the results were thoroughly examined, 48 articles were selected to be included in this narrative review (Table 1). A flowchart of the article selection process is represented in Figure 1.

## 3. Results and Discussion

### 3.1. Renal Cell Carcinoma

Renal cell carcinoma (RCC) accounts for 3% of all cancers, with the highest incidence occurring in Western countries: in 2020, 431,288 new cases of RCC were estimated globally, 138,611 of them in Europe [8,9,10]. RCC takes the lead as the primary malignant tumour arising in the kidneys, accounting for approximately 85–90% of all malignant renal tumours [11].

This cancer is categorized into 11 subtypes based on the 2004 World Health Organization (WHO) histologic classification of RCC. Among these subtypes, clear-cell RCC (ccRCC) prevails, representing about 70% of RCC cases. Following ccRCC is papillary RCC (pRCC), accounting for 10–15%, and chromophobe RCC (chRCC), constituting less than 5% of RCC cases [12].

These distinct subtypes of RCC exhibit varying biological behaviours, prognoses, and treatment pathways. Currently, a range of diagnostic techniques, including computed tomography (CT), Doppler ultrasound, magnetic resonance imaging (MRI), and angiography, are commonly employed to distinguish among the different RCC subtypes and differentiate them from other lesions like angiomyolipoma, oncocytoma, benign complex renal cysts, and others [13,14].

The diagnosis primarily hinges on identifying distinct imaging characteristics, yet the typical imaging patterns of an RCC subtype or a benign lesion might not always be evident. Additionally, there can be overlapping imaging features among RCC subtypes and certain benign lesions. For instance, both oncocytoma and chRCC might exhibit or lack a spoke-wheel-like enhancement in the central tumour region. Similarly, both pRCC and chRCC might display less intense, uniform, and pseudo-capsular enhancement in two-phase enhancement images. A hypovascular pRCC in the sole nephrographic phase might appear indistinguishable from a dense renal cyst. Consequently, distinguishing between various RCC subtypes and benign lesions through conventional CT imaging, or even by combining CT and MR, can pose significant challenges [15].

Quantitative iodine density imaging analysis (qIDI) is the most used technique to help distinguish between malignant and non-malignant renal lesions and also among different RCC subtypes [16,17,18,19,20,21,22].

Salameh JP and team’s meta-analysis [16] emphasizes that Dual-Energy Computed Tomography (DECT) with quantitative iodine density imaging (qIDI) exhibits sensitivity and specificity surpassing 95% when evaluating renal masses. This technique shows promise as an alternative to the standard CT approach. Notably, DECT’s ability to correct beam-hardening effects from iodine enables the precise identification of artificial attenuation increases in renal cysts, known as pseudo-enhancement—a limitation often encountered in traditional CT, especially with small and endophytic renal masses.

Interestingly, there was no significant difference in the accuracy between DECT using iodine quantification and Contrast-Enhanced CT (CECT) employing Hounsfield unit measurements. Similarly, no notable disparity was found between the dual-source DECT technique and the rapid kilovoltage switching method. However, it is important to approach these findings cautiously due to the limited number of studies included, some of which carry a high risk of bias. Larger-scale investigations are necessary to thoroughly assess DECT’s potential to replace conventional CT in clinical practice and determine its incremental advantages.

Wang D. and Colleagues [17] emphasize that qIDI in combination with the spectral analysis of the CT value of monochromatic images has the potential to enhance the diagnostic accuracy when distinguishing between pRCC and chRCC. In their research, they observed that during the cortical phase and parenchymal phase, the CT values and the slope of the spectrum curve for chRCC were notably higher than those for pRCC. This discrepancy was particularly pronounced at lower energy levels (40–70 keV), with a sensitivity of 76.5% and a specificity of 100% for differentiating between pRCC and chRCC.

Manoharan and colleagues [18] confirmed that single-acquisition triple-bolus Dual-Energy Computed Tomography (DECT) is non-inferior to triple-phase CT, displaying a similar diagnostic accuracy while significantly reducing the radiation exposure. This suggests that the triple-bolus DECT protocol could potentially become the preferred method for assessing renal masses, providing diagnostic, morphological, and functional insights while minimizing the radiation risk. In a subsequent study by the same group [19], they demonstrated that the quantitative iodine density imaging (qIDI) metrics derived from the triple-bolus DECT protocol strongly correlate with perfusion CT parameters like blood flow and blood volume, and moderately correlate with the vascular permeability. Furthermore, they emphasize a notable correlation between the permeability and iodine metrics. These findings align well with the fact that an increased vascular endothelial growth factor expression often results in a heightened permeability in tumour blood vessels. Importantly, the triple-bolus DECT technique involves approximately 15 times less radiation exposure compared to perfusion CT.

Dai and co-authors [20] further highlight the utility of quantitative iodine density imaging (qIDI) during a single nephrographic phase for distinguishing various RCC subtypes before surgery. Their study revealed a statistically significant contrast in iodine levels between ccRCC and both pRCC and chRCC. However, no significant distinctions in iodine levels were observed between pRCC and chRCC, or between high-grade and low-grade clear cell RCC, irrespective of the quantification method employed.

Zhang and colleagues [23] successfully correlated the iodine and water concentration using DECT with the microvascular density of RCC, demonstrating a correlation between the spectral parameters and neoangiogenesis.

Obmann MM et al. [21] suggested optimized thresholds for qIDI to identify the enhancement in incidental renal lesions upon single-phase DECT with sensitivity and specificity levels of up to 87.5% and 94.6%, respectively.

According to Udare et al. [22], qIDI proved highly accurate in distinguishing between cc-RCC and pRCC. To reduce the overlap with other types of tumours, the differentiation of clear cell RCC from other highly enhancing tumours could potentially be improved with the use of fat-specific images. In their research, the iodine concentration was notably higher in ccRCC compared to pRCC, but not significantly different from other tumours. In terms of intra-tumoural fat, they observed its presence in 36.0% (9/25) of ccRCC cases, 9.1% (1/11) of pRCC cases, and not in any other tumours. An iodine concentration of ≥3.99 mg/mL yielded an AUC along with a sensitivity/specificity of 0.88 (95% CI 0.76–1.00) and 92.31%/82.40%, respectively, for diagnosing ccRCC. Conversely, an iodine concentration of ≤2.5 mg/mL achieved an AUC and sensitivity/specificity of 0.99 (0.98–1.00) and 100%/100%, respectively, for diagnosing pRCC. For the presence of intra-tumoural fat, the AUC was 0.64 (95% CI 0.53–0.75) with sensitivity/specificity at 34.6%/93.8% for diagnosing cc-RCC. By employing a logistic regression model that combined iodine concentration and the presence of fat, the AUC increased to 0.91 (95% CI 0.81–1.0) with a sensitivity/specificity of 80.8%/93.8% for diagnosing cc-RCC.

Effective atomic number maps (Z-maps) are a different DECT output that was used by Mileto A. et al. [24] to differentiate non-enhancing renal cysts, including hyperattenuating cysts, from enhancing masses. This approach could prove valuable in situations where a comprehensive CT protocol for renal mass assessment is unavailable. In their investigation, they identified statistically significant discrepancies in the mean dual-energy effective atomic numbers between non-enhancing renal cysts and enhancing masses (8.13 ± 0.42 Z-eff for cysts vs. 9.37 ± 0.74 Z-eff for masses). Substantial differences in effective atomic numbers were observed when comparing Bosniak 2 renal cysts (8.24 ± 0.4 Z-eff) to enhancing renal masses, subsequently histologically confirmed ccRCC (9.64 ± 0.7 Z-eff), or pRCC (8.63 ± 0.4 Z-eff).

In cases with a confirmed histopathological diagnosis of an enhancing renal mass, significant discrepancies in Z-effective values were observed between RCC (9.24 ± 0.76 Z-effective) and other solid-enhancing masses like oncocytomas (9.71 ± 0.73 Z-effective). Importantly, dual-energy effective atomic numbers demonstrated significant distinctions in the comparison of clear cell (9.9 ± 0.68 Z-eff) and nonclear cell (8.64 ± 0.48 Z-eff) RCCs. There were no substantial differences in the dual-energy effective atomic numbers between ccRCC (9.64 ± 0.68 Z-eff) and solid-enhancing tumours other than RCC (9.71 ± 0.5 Z-eff). However, solid renal tumours other than RCC (e.g., oncocytomas) (8.7 ± 0.48 Z-eff) exhibited significant divergence from nonclear cell RCC subtypes (9.71 ± 0.5 Z-eff). With a Z-eff threshold of 8.36, the area under the curve (AUC) for distinguishing non-enhancing renal cysts from enhancing solid renal masses was 0.92 (95% CI, 0.89–0.94), with a 90.8% sensitivity, and 85.2% specificity, and an overall diagnostic accuracy of 86.6%. The overall diagnostic accuracy for discrimination among the different solid renal mass types varied: RCC versus other solid renal tumours (59.4%); solid renal tumours other than RCC versus nonclear cell subtypes of RCC (88.3%); clear cell RCC versus nonclear cell subtypes of RCC (82.5%); clear cell RCC versus solid renal tumours other than RCC (58.3%) [25].

**Figure 2:**
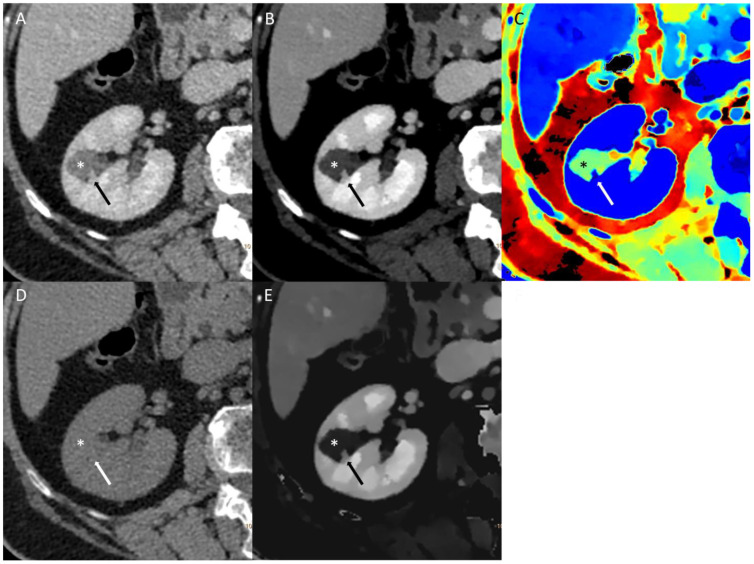
Conventional multi-energetic CT image in portal venous phase (**A**) of a small dense renal cyst (*) with a millimetric polypoid component (arrow). The cyst has a 49.2 HU density, thus barely visible in this image, so it is impossible to distinguish between a dense cyst and a hypovascular renal lesion. The 40 KeV Virtual Monoenergetic Image (**B**) shows poor changes in the enhancement of the cystic component and a good increase in the density of the polypoid component. Z-effective maps image (**C**) shows low atomic numbers within the cystic component and high numbers in the polypoid component. Virtual Non-Contrast image (**D**) shows no difference in the densitometry of the cystic component. Iodine density imaging (**E**) shows no iodine concentration in the cystic component and iodine concentration in the polypoid component. The lesion was classified as a class III Bosniak renal cyst, and afterwards, a multidisciplinary meeting was decided on for follow-up (mainly for dimensions). A control CT scan at 12 months (not shown) demonstrated no differences.

In the study by Bucolo GM and colleagues [25], the reliability of VNC images for evaluating renal masses was investigated in comparison to true non-contrast (TNC) images. The study involved the assessment of attenuation values and standard deviations by drawing regions of interest on both TNC and VNC images, which were reconstructed from corticomedullary (VNCc) and nephrographic (VNCn) phases. The results revealed differences in attenuation values of 74%, 18%, 5%, and 3% for TNC-VNCc and 74%, 15%, 9%, and 2% for TNC-VNCn. A Wilcoxon signed-rank test confirmed the equivalence of attenuation values between TNC and VNC images. Moreover, the diagnostic performance of VNC images in depicting kidney simple cysts remained high compared to TNC, with VNCc-AUC at 0.896 and VNCn-AUC at 0.901, while TNC-AUC stood at 0.903. Overall, the study demonstrated a strong agreement between the VNC and TNC images when assessing renal lesions. Specifically, in 92% of cases for VNCc and 89% for VNCn, the difference in attenuation values was less than 10 Hounsfield units (HUs) compared to TNC. While minor discrepancies were observed in a minority of cases, emphasizing the diagnostic value of TNC images, especially for the initial characterization of indeterminate renal masses, the VNC algorithm proved to be a reliable alternative in subsequent examinations. This approach offers significant benefits to patients in terms of radiation dose reduction [25]. An example of the spectral imaging of a challenging renal lesion can be found in Figure 2.

Notably, the initial reports on spectral CT and radiomics are starting to appear in the literature; however, they are demonstrating conflicting results [26,27].

Partial nephrectomy and enucleoresection such as percutaneous thermal ablation are the cornerstones of therapy for small kidney lesions [28,29]. Interestingly, we found no report inherent in post-surgical changes and only one report after percutaneous radiofrequency ablation changes, demonstrating the excellent diagnostic performance (sensitivity 100%; specificity 91.5%) of iodine overlay images for predicting local tumour progression [30].

### 3.2. Polycystic Kidney Disease

Autosomal Dominant Polycystic Kidney Disease (ADPKD) and Acquired Polycystic Kidney Disease (APKD) stand out as the most prevalent forms of polycystic kidney disorders (PKD) among adults, accounting for 3.06 cases per 100,000 person-years [31].

This genetic abnormality triggers the development of numerous bilateral renal cysts, with the potential for cysts to manifest in other organs such as the liver and pancreas. These cysts progressively multiply in number and expand in size throughout the individual’s lifetime. Consequently, the kidneys can attain a considerable size, leading to a noticeable mass effect. Due to the constant growth of cysts, they consistently encroach upon and replace the normal kidney tissue, thereby inducing a decline in kidney function. Over time, this process can culminate in end-stage renal disease (ESRD), necessitating either dialysis or kidney transplantation. Moreover, ADPKD inherently heightens the susceptibility to malignancies, especially renal cell carcinoma [32,33].

Arndt et al. [34] state that DECT significantly enhances the identification of malignancies in patients with PKD, simultaneously reducing the radiation exposure by eliminating the need for a true unenhanced phase given the possibility to obtain VNC imaging.

In patients with PKD, the crucial diagnostic marker for detecting malignancies involves assessing the contrast enhancement of potential masses. Traditionally, this assessment demanded meticulous comparisons of Hounsfield units between pre- and post-contrast scans, a method susceptible to challenges with side-by-side image analysis. Dual-energy CT introduces a solution by enabling the direct visualization of mass and cystic lesion enhancement through colour-coded representations. Additionally, it allows for the direct measurement of iodine concentration within the tissues (IDI) in contrast-enhanced scans without the need for an additional non-contrast scan [35].

Radiologists face a challenging task when evaluating the CT scans of polycystic kidney patients, involving the comparison of numerous cysts in both unenhanced and contrast-enhanced phase images. It is crucial to assess the iodine uptake in suspicious renal lesions to differentiate between malignant tumours and haemorrhagic or complex cysts. Many patients, especially those with ADPKD, present enlarged kidneys containing over 50 cysts. Utilizing DECT, the colour-coded visualization of the iodine distribution in CT images aids radiologists in pinpointing the enhancement within multiple lesions. This accelerates the differentiation process between suspicious lesions and simple cysts, streamlining the workflow [34]. Standard dual-phase kidney CT scans acquire phases sequentially, which might result in misalignment or motion artefacts. In cases of multiple renal cysts, anatomical mismatches can hinder the determination of the enhancement in a single cystic lesion. DECT, conversely, relies on only one phase for differentiation, minimizing the anatomical mismatch concerns [34].

### 3.3. Urothelial Cancer

Urothelial cancer stands as the seventh most common cancer in Western Europe, with a worldwide age-standardized incidence rate of 9.5 for men and 2.4 for women (per 100,000 person-years [36]. Urothelial cancer often impacts individuals in their eighth decade. It typically arises in the urinary bladder and is primarily signalled by visible blood in the urine (macroscopic haematuria), with bladder cancer manifesting in approximately 12–20% of these cases [37,38].

Research by Hansen et al. [39] proposes that employing DECT with VNC and IDI assessment holds promise as a valuable diagnostic tool for urothelial tumours. In their investigation, urothelial tumours were successfully identified in arterial phase series, 8 min nephrographic-excretory phase (split bolus), and a combination of both series with a sensitivity of 91.9% (95% CI, 78.1–98.2%), 83.4% (68.0–93.8%), and 97.3% (85.8–100%), respectively. Urothelial tumours exhibited more pronounced virtual enhancement and higher iodine concentration compared to lesions of different origins. Different threshold values for iodine concentration, spanning from 0.5 to 3.5 mg I/mL, underwent testing to pinpoint the most effective values for differentiating urothelial tumours from lesions originating from various histologic sources. The optimal sensitivity, reaching 91.9%, was attained using a threshold value of 1.0 mg I/mL or higher for urothelial tumours, whereas the greatest specificity, at 92.3%, was achieved with a threshold of 3.0 mg I/mL or higher.

In a separate study, Park et al. [40] reported sensitivities of 89–92% for the detection of urothelial cancer using 70 s (portal venous) phase MDCT images. Metser et al. [41] achieved a sensitivity of 89.3% for bladder cancer lesions using 60 s “urothelial-phase” images, compared to 70.5% with 5 min nephrographic-excretory phase images. Based on these findings, a single urothelial-phase MDCT protocol was proposed by the authors for high-risk patients [39].

Implementing these findings in clinical practice could help reduce the need for invasive endoscopic procedures, limiting them to cases where they are truly necessary. Additionally, the study indicates that VNC images could replace preliminary true unenhanced series, streamlining the DECT protocol into two phases [39].

**Figure 3:**
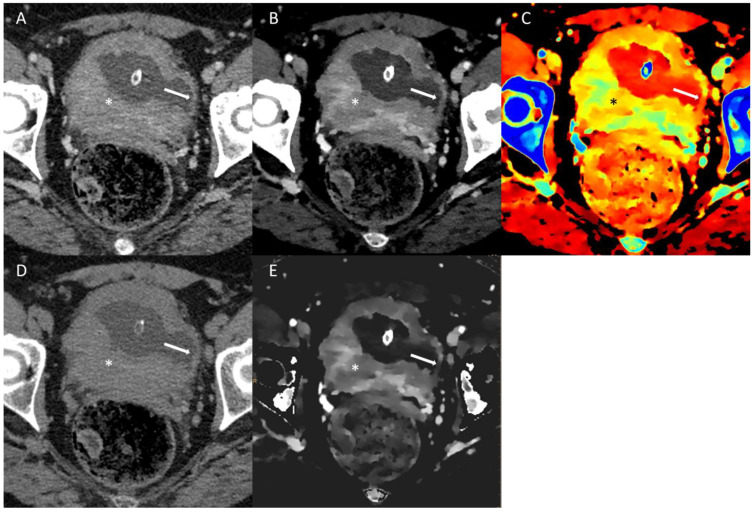
Conventional multi-energetic CT image in portal venous phase (**A**) of bladder cancer (*) in comparison with a small area of the healthy bladder wall (arrow). Virtual monoenergetic image (**B**) shows poor changes in the healthy bladder wall and inhomogeneous enhancement of the cancer. Z-effective maps image (**C**) shows atomic numbers within the healthy bladder wall and high numbers in cancer. Virtual Non-Contrast image (**D**) shows poor difference in the densitometry of the healthy bladder wall. Iodine density imaging (**E**) shows poor iodine concentration in the healthy bladder wall and high iodine concentration in the cancer.

An example of spectral imaging of a bladder lesion can be found in Figure 3.

In a study conducted by Zopfs D et al. [42], the researchers examined the use of low-keV virtual monoenergetic imaging reconstructions in patients with urothelial carcinoma during the excretory phase of spectral dual-energy CT scans. The study assessed various parameters, including attenuation, image noise, signal-to-noise ratio (SNR), and contrast-to-noise ratio (CNR), in both venous and excretory phase CT scans, as well as in excretory phase virtual monoenergetic images (VMIs) ranging from 40 to 70 keV. Measurements were taken using regions of interest (ROIs) in multiple regions, including the urothelial carcinoma, liver, pancreas, renal cortex, subcutaneous fat, renal vein/artery, portal vein, urinary bladder wall, lymph nodes, and prostate/uterus. The results of the study indicated that compared to the venous phase CT scans, excretory phase VMI at 40 keV exhibited a higher attenuation and SNR (*p* < 0.001) in most regions, except the liver parenchyma, where they were comparable. The image noise showed no significant difference between venous phase CT and excretory phase VMI at 40 keV (*p*-range: 0.08–1.00), except in the liver, portal vein, and renal artery, where it was lower in VMI at 40 keV (*p* < 0.05). Furthermore, the CNR between urothelial carcinoma and the surrounding bladder wall was significantly higher in the excretory phase VMI at 40 keV compared to the venous phase CT. The subjective assessments of vessel contrast and the delineation of primary tumours and distant metastases received equivalent or higher Likert scores for the excretory phase VMI at 40 keV compared to the venous phase CT. The authors conclude that for evaluating urothelial carcinoma, the use of virtual monoenergetic excretory phase images at 40 keV obtained using spectral dual-energy CT could be a viable option for maintaining both subjective and objective image quality, as seen in conventional venous phase images [42].

The most prevalent benign condition affecting men aged 50 to 70 is benign prostate hyperplasia (BPH) [43]. Severe BPH accompanied by intravesical protrusion can trigger bladder outlet obstruction, leading to symptoms resembling bladder cancer [44]. Traditional polychromatic CT facilitates the clear observation of the site, extent, and infiltration degree of bladder cancer, and it can effectively depict the signs of adjacent organ invasion and lymph node metastasis. Nonetheless, severe BPH can result in the protrusion of nodular, mass-like tissue extending upward to the base of the bladder [45,46]. Consequently, CT sometimes struggles to distinguish between bladder cancer and severe BPH also because of the similar CT numbers in traditional CT [47]. According to Chen et al. [48], dual-energy spectral CT imaging offers heightened sensitivity and specificity in distinguishing between bladder cancer and benign prostate hyperplasia. Their study demonstrated a statistically significant difference in CT values between the bladder cancer group and the BPH group across energy levels ranging from 40 to 90 keV, with the most pronounced difference observed at 40 keV. Moreover, the slope of the spectral Hounsfield unit (HU) curve for bladder cancer was notably steeper than that for BPH. Smaller differences in CT values were noted at higher energy levels. They also identified statistically significant differences in the Z-eff number and its peak value between the two groups.

Bladder cancer cells often display abnormal arrangements, characterized by cell nucleus anaplasia, whereas BPH is mainly marked by an increased density of smooth muscle cells. The distinct characteristics between bladder cancer and BPH were delineated by the higher atomic number and the more rapid changes in CT values as a function of photon energy (greater slope value of the spectral HU curve) observed in bladder cancer in their study. The study suggested that the slope of the spectral HU curve for bladder cancer was significantly higher than that for BPH; the bladder cancer Gemstone Spectral Imaging (GSI) curve exhibited a sharper trajectory than the BPH GSI curve, implying a higher attenuation in bladder cancer. Additionally, a statistically significant difference in Z-eff number was observed between bladder cancer and BPH. The measurement of CT values at 40 keV highlighted the most significant divergence between bladder cancer and BPH due to the accentuated attenuation difference of different materials caused by lower-energy photons [49]. By employing a CT value threshold of 73.4 HU at 40 keV, they achieved sensitivity and specificity of 77.0% and 82.5%, respectively, with an AUC of 0.817 for distinguishing bladder cancer from BPH.

In conclusion, spectral CT enables the multiparametric imaging of the urinary tract. CT value measurement on monochromatic images at 40 keV offers a high sensitivity and specificity in distinguishing posterior wall bladder cancer with intravesical protrusion from BPH [48].

### 3.4. Urolithiasis

Urolithiasis is a prevalent global issue with significant implications for health and survival, with a 5.2% lifetime prevalence [50]. Understanding the chemical composition of calculi at the time of diagnosis could contribute to better patient management [51]. Different types of urinary stones have varying degrees of fragility, influencing the suitability of surgical interventions. The capacity of single-energy CT to determine the chemical composition of urinary calculi is limited due to overlapping CT numbers among different types of calculi [52]. In contrast, DECT utilizes the alteration in attenuation of calculi between low- and high-energy spectra to differentiate their components [53]. Urinary calculi characterization can involve calculating the dual-energy ratio in dual-source DECT or determining the Z-eff using rapid-switching and spectral detector CT. These values are then compared with known chemical compositions to classify the calculi. DECT outperforms single-energy CT in accurately distinguishing uric acid calculi from non-uric acid calculi (90–100%) and in the finer classification of non-uric acid calculi [54,55,56,57,58,59]. A recent feasibility study suggests that using DECT characteristics to assess stone fragility holds promise in providing clinically relevant data that could influence surgical decisions [60]. In practical terms, DECT for urinary calculi characterization is typically necessary only during the initial scan of a patient with known or suspected urinary calculi.

A factor limiting the broader adoption of DECT for urinary calculi characterization is the potential misclassification of calculi with mixed compositions, which is common in the majority of cases, due to overlapping dual-energy characteristics. The advantage of DECT in CT urography lies in its ability to generate virtual unenhanced images from the contrast-enhanced phase, eliminating the need for a true unenhanced phase [61,62,63]. The accuracy of VNC imaging in detecting urinary calculi is influenced by both the size and attenuation of the calculi. It tends to decrease for smaller (<3 mm) and less dense (<400 HU) calculi [64,65]. The VNC images derived from the excretory phase of DECT urography have shown a sensitivity range of 53–87% for detecting urinary calculi [64]. However, the presence of dense contrast material in the excretory system can lead to beam hardening and increased noise in the virtual unenhanced images, potentially obscuring smaller, lower-attenuation calculi. The use of dual-source DECT with a tube combination of 100 kV and 140 kV, along with a tin filter, has been reported to mitigate this artefact [64]. Further validation of this approach could enhance the clinical utility of DECT urography.

**Figure 4:**
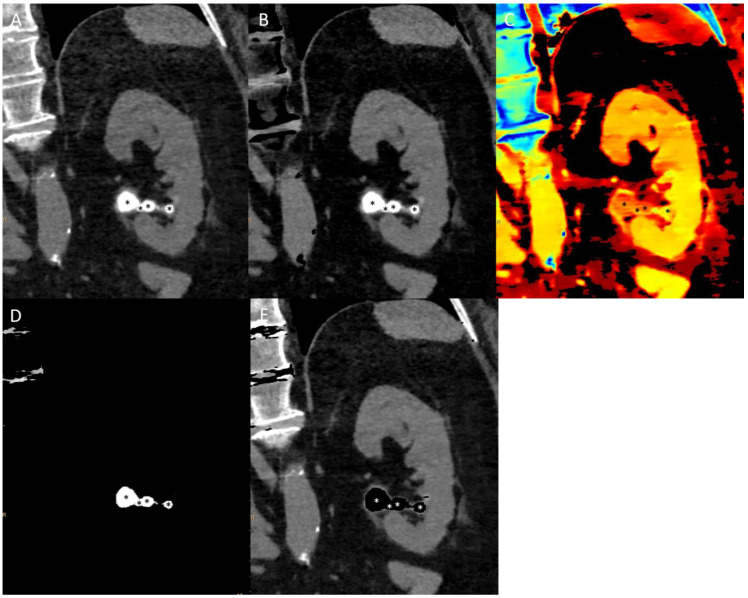
Non-contrast CT scan of uric acid kidney stones (*) in para-coronal reformations: conventional multi-energetic CT image (**A**), calcium suppression (**B**), Z-effective maps image (**C**), uric acid only image (**D**), and uric acid suppression image (**E**). Note the absence of suppression in B and the difference in Z-effective images between stones and spine bones, which are rich in calcium.

An example of spectral imaging characterization of a kidney stone can be found in Figure 4.

### 3.5. Lymph Nodes

Recently, numerous studies have validated the use of DECT quantitative parameters for evaluating metastatic lymph nodes (LNs) in different cancer types such as ovarian cancer [6], lung cancer [65,66], colorectal cancer [67], papillary thyroid cancer [68,69], and pancreatic ductal adenocarcinoma [70]. However, it is important to highlight the existing gap in the research pertaining to both metastatic and non-metastatic LNs in urogenital cancer patients, except for the cases related to prostate cancer [71].

Liu J et al. [66] conducted a retrospective analysis of 80 lymph nodes (LNs) in rectal cancer, comprising 57 non-metastatic LNs and 23 metastatic LNs, taken from 42 patients with pT1-T2 rectal cancer. Their findings revealed that all the quantitative parameters derived from DECT including IDI, Z-eff, λ (slope of the attenuation curve), normalized IDI (nIDI), and normalized Z-eff (nZ-eff) were different in the LN metastatic vs non-metastatic group. This observation aligned with previous research [72]. Several factors were considered to explain these results: (a) the iodine content reflects the tissue’s vessel permeability and blood volume and factors detectable via quantitative DECT parameters obtained from the portal phase, such as IDI, Z-eff, nIDI, nZ-eff, and λ [73,74]; (b) malignant LNs were found to have a lower vessel density compared to benign LNs [75]; (c) malignant LNs often exhibit central necrosis due to an abundance of infiltrated tumour cells with insufficient blood supply, resulting in hypodensity on imaging. The researchers identified nZ-eff as an independent risk factor for lymph node metastasis, with an area under the curve (AUC) of 0.870, offering a sensitivity of 82.5% and specificity of 82.6% for detecting metastatic LNs. In their study, the highest diagnostic performance and sensitivity for evaluating metastatic LNs, reaching 100%, were achieved by combining nZ-eff with the short-axis diameter of the LN. In conclusion, the spectral parameters derived from DECT were found to enhance the preoperative accuracy of diagnosing metastatic LNs in patients with pT1–2 rectal cancer; the multi-parameter regression model, which combines nZ-eff with the short-axis diameter, demonstrated the highest diagnostic performance [67].

Liu H et al. conducted a study [76] in which they analyzed 152 LNs in rectal cancer, consisting of 92 non-metastatic LNs and 60 metastatic LNs, using radiological–pathological correlation. Their findings revealed that the mean short-axis diameter of metastatic LNs was significantly larger than that of non-metastatic LNs (7.28 ± 2.28 mm vs. 4.90 ± 1.64 mm, *p* < 0.001). Additionally, the mean nIDI value for metastatic LNs was significantly lower than that for non-metastatic LNs (0.24 ± 0.08 vs. 0.34 ± 0.21, *p*: 0.001 in the arterial phase; 0.47 ± 0.18 vs. 0.64 ± 0.17, *p* < 0.001 in the portal venous phase). By combining nIDI (PP) with the short-axis diameter, they were able to improve the overall accuracy to 82.9%. Therefore, the authors suggest that by utilizing the nIDI value in the portal venous phase in conjunction with the conventional size criteria, DECT imaging has the potential to distinguish metastatic lymph nodes from non-metastatic ones in rectal cancer [76].

Furthermore, Chen WB et al. conducted a study [77] investigating the diagnostic utility of spiral CT Energy spectrum imaging in detecting lymph node metastasis in colorectal cancer. In their research, during both the arterial and venous phases, they observed that the single-energy CT values ranging from 40 to 140 keV were significantly higher in the non-metastatic group compared to the metastatic group (all *p* < 0.05). Additionally, they found that the parameter values including IDI, nIDI, λ (the slope of the energy spectrum curve), and Z-eff were also higher in the non-metastatic group than in the metastatic group (all *p* < 0.05). Further analysis using receiver operating characteristic (ROC) curves revealed that in the arterial phase, the single-energy CT value at 50 keV had the highest AUC of 0.889. Among the energy spectrum parameters (IDI, nIDI, λ, and Z-eff), nIDI demonstrated the best diagnostic efficiency, with an AUC of 0.873. The combination of nIDI and λ achieved the highest AUC of 0.885 when these energy spectrum parameters were combined. In the venous phase, the single-energy CT value at 60 keV had the highest AUC of 0.853. Among the energy spectrum parameters, λ displayed superior diagnostic efficiency, with an AUC of 0.822. Combining nIDI, λ, and Z-eff yielded the highest AUC of 0.840 when considering these energy spectra together. In conclusion, the authors suggest that the energy spectrum CT imaging parameters are effective in evaluating the presence of LN metastases in rectal cancer [77].

Combining DECT with the generation of iodine decomposition images provides a means to semi-quantitatively assess the relative iodine concentrations within lesions. This computed information holds promise in differentiating between inflammatory and metastatic LNs. The research carried out by Tawfik et al. [78], focusing on cervical adenopathy with metastatic squamous cell cancer, revealed that while both reactive and metastatic lymph nodes exhibit enhancement, the calculated iodine concentration (expressed in milligrams per millilitre) is notably lower for metastatic nodes (mean ± SD, 2.34 ± 0.45 mg/mL) when compared to benign nodes. DECT has shown its capability to enhance the precision of nodal cancer staging as lymph nodes may be more clearly visible in iodine-enhanced images.

In a recent study led by Pan et al. [79], the investigators examined the iodine concentration (expressed as milligrams per millilitre) in LNs, normalized to the iodine concentration in the aorta (normalized iodine concentration = iodine concentration in the lesion/iodine concentration in the aorta). This normalization was carried out to discriminate between benign and malignant LNs. The study unveiled distinct normalized iodine concentrations between benign and malignant lymph nodes during both the arterial and portal venous phases (mean ± SD, 0.22 ± 0.09 vs. 0.13 ± 0.06 mg/mL and 0.47 ± 0.14 vs. 0.30 ± 0.12 mg/mL, respectively).

In the context of metastatic lymph nodes in urogenital tumours, Lennartz and colleagues [71] conducted a study to assess the relationship between iodine quantification using spectral detector CT and PSMA PET/CT. Their research revealed that metastatic lymph nodes identified by PSMA PET/CT exhibited a higher iodine concentration (IC) than non-metastatic nodes (mean ± SD, 1.9 ± 0.6 mg/mL vs. 1.5 ± 0.5 mg/mL, *p* < 0.05), resulting in an area under the curve (AUC) of 0.72 and sensitivity/specificity of 81.3%/58.5%. Additionally, the mean short-axis diameter of metastatic LNs was larger than that of non-metastatic nodes (mean ± SD, 6.9 ± 3.6 mm vs. 5.3 ± 1.3 mm; *p* < 0.05). Using a short-axis diameter threshold of 1 cm resulted in a sensitivity/specificity of 12.8%/99.0%. They noted a significant yet weak positive correlation between SUVmax and IC (rs = 0.25; *p* < 0.001). Based on these findings, the authors concluded that while spectral detector CT-derived IC is indeed significantly higher in cases of suspected metastatic spread, it possesses limited value for detecting metastatic lymph nodes in prostate cancer, particularly considering the clinical accuracy required. Therefore, they recommend the use of PSMA PET/CT for diagnosing LN metastases in prostate cancer.

Additionally, in a separate study [80], the same research group confirmed that VMI maintains diagnostic quality in abdominal spectral detector CT with reduced contrast media. They noted that VMI at 40 keV effectively counteracts the deterioration of contrast in contrast media-reduced abdominal spectral detector CT, facilitating accurate diagnostic assessment. Particularly noteworthy is that SDCT-derived VMI at 40 keV provides sufficient visualization of vessels, organs, and lymph nodes even when there is a significant reduction in CM [80].

However, further dedicated studies on metastatic LNs are required to validate the findings regarding DECT in urogenital tumours.

### 3.6. Limitations

This work suffers from intrinsic limitations in terms of objectivity, the completeness of the literature search, and interpretation of the findings of all the narrative reviews. We tried to be the most objective and exhaustive as we could, both by conducting independently the screening of the initial database research by two different authors and also by adding citations also from the bibliography of the findings of the original search. Moreover, unfortunately, poor data are presented in literature regarding nodal secondarisms from urological primaries presently. To properly discuss the topic, we added information about lymph nodes’ involvement in different cancers.

## 4. Conclusions

In conclusion, Dual-Energy Computed Tomography (DECT) with its various advanced techniques, including Virtual Non-Contrast (VNC), effective atomic number (Z-eff) calculation, Z-maps, and Iodine Density Index (IDI), holds great promise in the diagnosis and management of urogenital tumours, given its multiparametricity. The potential for this technology to provide better lesion characterization, improved staging accuracy, and more precise treatment response assessment is evident.

However, it is essential to emphasize that, despite the progress made so far, further studies are needed to establish the clinical utility of DECT in this context. The requirement for enrolling a larger number of patients in clinical trials is crucial to comprehensively evaluate the effectiveness of these techniques and their applicability in routine clinical practice.

Furthermore, it is important to consider the ongoing evolution of the DECT technology and its integration with other diagnostic and therapeutic tools in the field of urogenital oncology.

Ultimately, DECT represents a promising frontier in the diagnosis and management of urogenital tumours, reducing the gap with multiparametric MR imaging, but its full potential can only be realized through additional research efforts and multidisciplinary collaboration to translate these innovations into everyday clinical practice.

**Table 1:** A comprehensive table of the presented studies.

Author	Journal	Year	Pathology	Technique	Study Design	
Salameh JP [16]	AJR	2019	Renal Cell Carcinoma	qIDI	Systematic Review and Meta-Analysis	\
Wang D. [17]	Acta Radiol	2020	Renal Cell Carcinoma	qIDI	Retrospective	in vivo
Manoharan D. [18]	AJR	2018	Renal Cell Carcinoma	qIDI	Prospective	in vivo
Manoharan D. [19]	AJR	2020	Renal Cell Carcinoma	qIDI	Prospective	in vivo
Dai C. [20]	Abdom Radiol (NY)	2018	Renal Cell Carcinoma	qIDI	Retrospective	in vivo
Obmann M.M. [21]	Abdom Radiol (NY)	2020	Renal Cell Carcinoma	qIDI	Retrospective	in vivo
Udare A. [22]	Eur Radiol	2020	Renal Cell Carcinoma	qIDI	Prospective	in vivo
Zhang B. [23]	BMC Cancer	2021	Renal Cell Carcinoma	IDI	Retrospective	in vivo
Mileto A. [24]	AJR	2017	Renal Cell Carcinoma	Z-eff; Z-maps	Retrospective	in vivo
Bucolo G.M. [25]	JCM	2023	Renal Cell Carcinoma	VNC	Retrospective	in vivo
Han D. [26]	Clin Radiol	2021	Renal Cell Carcinoma	VMI; Radiomics	Retrospective	in vivo
Ding Y. [27]	Acta Radiol	2022	Renal Cell Carcinoma	VMI; IDI; Radiomics	Retrospective	in vivo
Park S.Y. [30]	Eur J Radiol	2014	Renal Cell Carcinoma	VMI; VNC	Retrospective	in vivo
Arndt N. [34]	Eur Radiol	2012	Polycystic Kidney Disease	VMI; VNC; qIDI; IDI	Prospective	in vivo
Graser A. [35]	Invest Radiol	2010	Polycystic Kidney Disease	VNC; IDI	Prospective	in vivo
Hansen C. [39]	AJR	2014	Urothelial Carcinoma	VNC; qIDI	Retrospective	in vivo
Zopfs, D. [42]	Eur J Radiol	2019	Urothelial Carcinoma	VMI	Prospective	in vivo
Chen A. [48]	Medicine (Baltimore)	2016	Urothelial Carcinoma	VMI; Z-eff	Retrospective	in vivo
Mansouri M. [53]	Curr Probl Diagn Radiol	2015	Urolithiasis	VMI; qIDI	Review	\
Hidas G. [54]	Radiology	2010	Urolithiasis	VMI	Prospective	in vivo
Leng S. [55]	AJR	2015	Urolithiasis	VMI	Prospective	in vivo
Manglaviti G. [56]	AJR	2011	Urolithiasis	VMI	Retrospective	in vivo
Primak A.N. [57]	Acad Radiol	2007	Urolithiasis	VMI	Prospective	ex vivo
Stolzmann P. [58]	Abdom Imaging	2010	Urolithiasis	VMI	Prospective	in vivo
Wisenbaugh E.S. [59]	Urology	2014	Urolithiasis	VMI	Prospective	ex vivo
Ferrero A. [60]	Acad Radiol	2016	Urolithiasis	VMI	Prospective	ex vivo
Moon J.W. [61]	Br J Radiol	2012	Urolithiasis	VNC	Retrospective	in vivo
Takahashi N. [62]	AJR	2008	Urolithiasis	VMI; VNC; IDI	Prospective	ex vivo
Takahashi N. [63]	Radiology	2010	Urolithiasis	VMI; VNC	Retrospective	in vivo
Mangold S. [64]	Radiology	2012	Urolithiasis	VNC	Retrospective	in vivo
Zorzetto G. [6]	Eur Radiol Exp	2022	Lymph nodes	VMI; Z-eff	Retrospective	in vivo
Hu X. [65]	J Comput Assist Tomogr	2021	Lymph nodes	VMI; VNC; IDI	Retrospective	in vivo
Nagano H. [66]	AJR	2022	Lymph nodes	IDI	Retrospective	in vivo
Liu J. [67]	Abdom Radiol (NY)	2023	Lymph nodes	IDI; qIDI; Z-eff; λ	Retrospective	in vivo
Jin D. [68]	Front Oncol	2022	Lymph nodes	VMI; IDI	Retrospective	in vivo
Yoon J. [69]	PLoS One	2021	Lymph nodes	Z-eff; IDI; λ	Retrospective	in vivo
An C. [70]	Eur J Nucl Med Mol Imaging	2022	Lymph nodes	VMI	Prospective	in vivo
Lennartz S. [71]	Clin Nucl Med	2021	Lymph nodes	IDI	Retrospective	in vivo
Rizzo S. [72]	Eur Radiol	2018	Lymph nodes	VMI; IDI	Retrospective	in vivo
Yang Z. [73]	AJR	2019	Lymph nodes	Z-eff	Prospective	in vivo
Fan S. [74]	Eur J Radiol	2017	Lymph nodes	VMI; qIDI	Retrospective	in vivo
Liu H. [76]	Eur J Radiol	2015	Lymph nodes	VMI; qIDI	Retrospective	in vivo
Chen W.-B. [77]	Int J Colorectal Dis	2022	Lymph nodes	VMI; qIDI; Z-eff; λ	Retrospective	in vivo
Tawfik A.M. [78]	Eur Radiol	2014	Lymph nodes	IDI	Retrospective	in vivo
Pan Z. [79]	PLoS One	2013	Lymph nodes	VMI; qIDI	Prospective	in vivo
Lennartz S. [80]	Br J Radiol	2020	Lymph nodes	VMI	Retrospective	in vivo

## Data Availability

No new data were created or analyzed in this study. Data sharing is not applicable to this article.

## Abbreviations

ADPKD	Autosomal Dominant Polycystic Kidney Disease
APKD	Acquired Polycystic Kidney Disease
AUC	Area Under the Curve
BPH	Benign Prostate Hyperplasia
ccRCC	Clear-cell Renal Cell Carcinoma
chRCC	Chromophobe Renal Cell Carcinoma
CT	Computed Tomography
DECT	Dual-Energy Computed Tomography
GSI	Gemstone Spectral Imaging
IDI	Iodine Density Index
λ	Slope of the Attenuation Curve
LNs	Lymph Nodes
MRI	Magnetic Resonance Imaging
PKD	Polycystic Kidney Disorders
pRCC	Papillary Renal Cell Carcinoma
qIDI	Quantitative Iodine Density Imaging Analysis
RCC	Renal Cell Carcinoma
ROC	Receiver Operating Characteristic
VMI	Virtual Monoenergetic Imaging
VNC	Virtual Non-Contrast
Z-eff	Effective Atomic Number
Z-Maps	Maps of Prevalent Atomic Number
